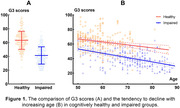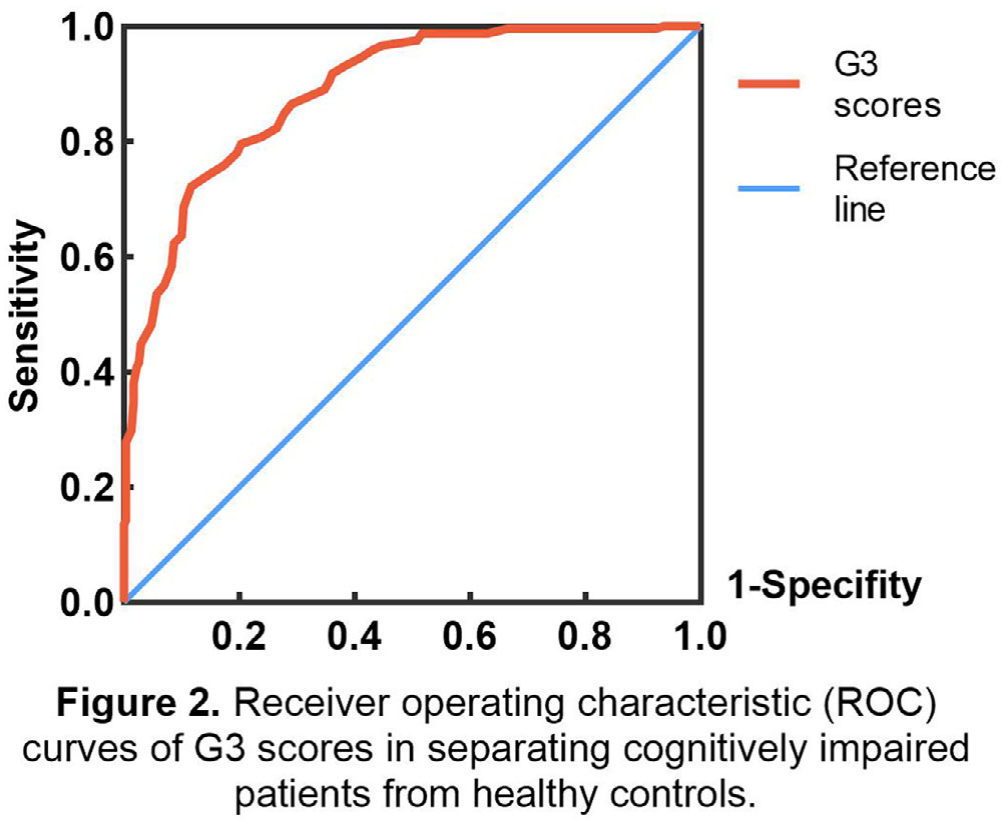# Validation Study of A Three‐Minute Game‐Based Cognitive Screening Tool For Detecting Cognitive Impairments In Chinese Older Adults

**DOI:** 10.1002/alz.094255

**Published:** 2025-01-09

**Authors:** Jingnan Wu, Yatian Li, Zhixing Zhou, Huanhuan Xia, Nan Chen, Qihao Guo

**Affiliations:** ^1^ Shanghai Bestcovered Limited, Shanghai China; ^2^ Shanghai Sixth People’s Hospital Affiliated to Shanghai Jiao Tong University School of Medicine, Shanghai China

## Abstract

**Background:**

Game‐based Cognitive Assessment – 3‐Minute Version (G3) is a WeChat mini‐program developed for the early screening of cognitive impairments in Chinese older adults. It consists of three game‐like digital cognitive tests, namely “Number Ordering”, “Species Sorting”, and “Gold Finding”. This study aims to assess the sensitivity, specificity, and diagnostic accuracy of G3 in detecting mild cognitive impairment (MCI) and early‐stage dementia in Chinese older adults.

**Method:**

Between February and December 2023, a total of 475 older adults (aged 50 to 89 years) with subjective cognitive complains were recruited. Participants underwent G3 assessment, neuropsychological assessments, neuroimaging, and blood tests. Among them, 230 individuals were diagnosed with cognitive impairments, including MCI and early‐stage dementia, while the remaining 245 older adults exhibited healthy cognition. We compared the G3 performance between the two groups. And the receiver operating characteristic (ROC) curve of G3 were evaluated with sensitivity and specificity calculated.

**Result:**

The average G3 scores (mean ± standard deviation) in the healthy cognition group (63.2±13.04) were significantly higher than impaired cognition group (41.3±12.50, p<0.001). In both groups, the mean G3 scores tended to decline with increasing age. Meanwhile, the decline was more significant in the impaired cognition group compared to the healthy cognition group (Figure 1). G3 demonstrated high diagnostic accuracy in detecting individuals with MCI or early‐stage dementia, with an area under the ROC curve (AUC) of 0.887 (95% confidence interval [CI] = 0.859‐0.916, Figure 2). The optimal cut‐off score for G3 was determined to be 59.5, with a sensitivity of 0.913 and specificity of 0.624.

**Conclusion:**

G3 WeChat mini‐program is a precise and user‐friendly screening tool that exhibits high sensitivity and diagnostic accuracy in detecting early‐stage cognitive impairment among community‐dwelling seniors. Further multicenter research is needed to verify the diagnostic value of G3 for different stages and types of cognitive impairments.